# The challenges experienced by patients in the early period after thyroidectomy and the effects on sleep quality

**DOI:** 10.1007/s00520-025-09483-w

**Published:** 2025-05-01

**Authors:** Gülseren Maraş Baydoğan, Yeliz Sürme, Songül Kütük Karasungur

**Affiliations:** 1 Erciyes University, Faculty of Health Sciences, Surgery Nursing, Kayseri, Türkiye; 2 Erciyes University, Medical Faculty Hospital, Otorhinolaryngology Clinic Nurse, Kayseri, Türkiye

**Keywords:** Thyroidectomy, Postoperative problems, Sleep quality

## Abstract

**Background:**

Thyroidectomy, a standard surgical procedure, can lead to various postoperative issues that can significantly affect a patient’s sleep quality. This study, addressing a topic of significant importance to thyroid surgery and patient care, engages healthcare professionals, researchers, and academics, providing crucial insights into the challenges faced by patients in the early period after thyroidectomy and their effects on sleep quality.

**Objective:**

The primary aim of this study was to investigate the prevalence of postoperative issues experienced by patients in the early period after thyroidectomy and their influence on sleep quality.

**Methods:**

This descriptive and prospective study, conducted with a sample of 104 thyroidectomy patients hospitalized in a university’s Ear, Nose, and Throat clinic between 2021 and 2023, employed a comprehensive set of tools for data collection. These tools included a questionnaire, Visual Analog Scale for pain and nausea, Symptom Severity Scale, and Richard Campbell Sleep Scale, ensuring a thorough investigation into the prevalence of postoperative issues and their influence on sleep quality.

**Results:**

The most frequently reported postoperative issues were pain, difficulty swallowing, sore throat, dry mouth, nausea-vomiting, tingling, hoarseness, Chvostek’s sign, tremors (tetany), Trousseau’s sign, and bleeding. Analysis of the pre-and postoperative Richard Campbell Sleep Scale scores revealed a significant decrease in sleep quality post-surgery, particularly in patients experiencing sore throat, difficulty swallowing, pain, Chvostek’s sign, and Trousseau’s sign.

**Conclusions:**

Our study has revealed that patients’ three most commonly reported problems after thyroidectomy are pain, difficulty swallowing, and sore throat. Importantly, these issues significantly impact sleep quality, underscoring the need for effective management to improve patient recovery. This study enhances our understanding of postoperative issues after thyroidectomy and inspires improved clinical practices in managing these challenges.

## Introduction

Thyroidectomy is one of the most common surgical procedures used to treat malignancies and impaired thyroid function. The surgical process is complex, ranging from simple removal of nodules to extensive removal of the thyroid gland [1]. The primary goal of thyroid surgery is to wholly or permanently control the disease and reduce morbidity. The advantages of thyroidectomy surgery include control of hyperthyroidism, treatment of thyroid nodules and carcinoma, prevention of ocular complications in Graves’ disease, protection from radioactive material exposure, and minimization of side effects of antithyroid drugs. The disadvantages include the need for lifelong thyroid hormone replacement and the risk of developing complications such as hypoparathyroidism, recurrent laryngeal nerve damage due to iatrogenic injury, hypocalcemia, and neck hematoma [2]. Most thyroidectomies are performed as elective surgery, allowing for comprehensive planning [2, 3]. Thyroidectomy remains the most commonly performed safe model of endocrine surgery worldwide, although postoperative complications can arise.

Daba et al. (2023) [4] reported that 29.4% of patients developed complications at the first follow-up after thyroidectomy, and 20.6% developed permanent complications at the 6-month follow-up. In another study, it was found that the majority of complications occurred before discharge [5]. Although complications such as mortality and surgical site infections are low after thyroidectomy, hypocalcemia (serum calcium level < 7.6 mg/mL), recurrent laryngeal nerve (RLN) injury, and cervical bleeding/hematoma are known to be the most significant postoperative complications [6]. It is reported that permanent hypocalcemia occurs in 7–51% of patients after surgery, RLN injury in 3–11% [7], and bleeding/hematoma in 12% [2, 6]. Severe effects of high rates of hypocalcemia can lead to cardiac arrhythmias, tetany, numbness, tingling, hyperparaesthesia, muscle contractures, seizures, and an increased risk of mortality. Therefore, it is recommended to start taking oral calcium and vitamin D during the perioperative period, mainly because it reduces the risk of hypocalcemia [8].

Recurrent laryngeal nerve injury can result in hoarseness or dysphonia. Typically, swallowing problems, particularly with liquids/solid foods, can also be observed [9, 10]. Postoperative bleeding/hematoma causes serious issues that are life-threatening and require urgent surgical decompression. While most hematomas clinically manifest in the postoperative period (within the first six hours), up to 25% occur within 6–24 h after the procedure [11]. Patients undergoing thyroidectomy are faced with various issues during the postoperative period, including pain, thirst, dry mouth, sore throat, nausea, vomiting, and dysphagia. Sore throat and dry mouth often occur due to intubation, while pain, nausea, and vomiting develop primarily due to the surgery itself and anesthesia-related factors [12].

These problems after surgery can adversely affect the patient's sleep quality [13, 14]. The prevalence of sleep disturbances in cancer patients is reported to be 33–40%, which is approximately twice the rate reported in the general population. Physiological and psychological burdens related to the patient’s condition and treatment can increase the risk of poor sleep quality. Results suggest an association between sleep disorders and an increased risk of thyroid cancer [14]. Similarly, quality sleep is reported to be a protective factor against thyroid cancer [15].

The rapid recovery protocols implemented by healthcare systems aiming to achieve the shortest possible postoperative discharge have increasingly emphasized the early detection, monitoring, and management of post-thyroidectomy outcomes and complications [16]. Detecting the problems experienced by patients in the early period can facilitate the management of the intervention process by identifying risk factors for complications, predicting complications, and intervening accordingly [5]. Effective management of this process can reduce preoperative and postoperative problems for patients, improve recovery, and enhance sleep quality.

This study is aimed at determining the incidence of problems experienced by patients in the early period after thyroidectomy and their impact on sleep quality.

## Method

### Population and sample of research

The research was conducted at the Ear, Nose, and Throat (ENT) service of the Erciyes University Health Application and Research Center. The descriptive and prospective study sample consisted of thyroidectomy patients hospitalized between May 2021 and May 2023. As a standard procedure, patients undergoing thyroidectomy were operated on under general anesthesia. There is no prophylactic pharmacological treatment available for nausea that occurs during thyroidectomy surgery at our university. Following thyroidectomy, patients are medically treated with ampicillin and sulbactam 2 × 1 or 3 × 1, dexketoprofen (2 × 1), and pantoprazole sodium sesquihydrate (1 × 1). Patients who do not report hypocalcemia, tingling sensations, or numbness and whose drainage output reduces to below 10 mL within 14 h for those with drains are discharged on average in 3–4 days. They are followed up at the outpatient clinic on the 15 th day after discharge.

The study, which focused on patients aged 18 and over who had undergone thyroidectomy surgery and were hospitalized in the ENT service, was conducted with a total of 104 patients. Notably, the power of the study was found to be 99% in the post-power analysis, a unique aspect of our research that underscores its robustness and reliability.

### Data collection tools

Data were collected using a descriptive characteristics form, a Postoperative Symptom Form, and The Richard Campbell Sleep Questionnaire.

### Descriptive characteristics form

The questionnaire form, which the researchers developed by scanning the literature [6, 11, 12], consists of questions about the sociodemographic data of the patients (such as age, gender, height, weight, marital, and employment status), chronic disease, surgery history, smoking, ASA score, surgery duration, calcium, and vitamin D levels.

### Postoperative symptom form

There are 11 symptoms, including postoperative pain, difficulty swallowing, sore throat, dry mouth, nausea-vomiting, tingling, hoarseness, tremor (tetany), bleeding, Chvostek’s sign, and Trousseau’s sign [6, 11, 12].

### The Visual Analog Scale (VAS)

The Visual Analog Scale (VAS), graded between 0 and 10 cm, assessed the severity of postoperative pain and nausea/vomiting. The patient indicated the severity of nausea/pain on this scale: 0 = no nausea/no pain, 1–3 = mild, 4–6 = moderate, and 7–10 = severe. The severity of postoperative sore throat, hoarseness, difficulty swallowing, and dry mouth was assessed using a symptom severity scale graded between 0 and 3. The scale is indicated as follows: 0 = none; 1 = mild; 2 = moderate; 3 = severe [12].

### The Richard Campbell Sleep Questionnaire

The Richard Campbell Sleep Questionnaire (RCSQ), developed by Richards (1987) [17], is a six-item scale that assesses the depth of nocturnal sleep, the time it takes to fall asleep, the frequency of awakenings, the duration of wakefulness upon awakening, sleep quality, and the level of ambient noise. The Turkish validity and reliability of the scale were conducted by Özlü and Özer (2015) [18]. Each item is evaluated on a chart ranging from 0 to 100 using the visual analog scale technique. The Cronbach’s α value of the scale was found to be 0.91. In this study, Cronbach’s α value was also found to be 0.91.

### Data collection

In the first stage, patients’ sociodemographic data were obtained through face-to-face interviews with the patients. In the second stage, the problems experienced in the first 24 h after the patients were admitted to the postoperative service were evaluated and recorded. The sleep scale was administered one day before discharge. The data collection process took an average of 15 min.

### Ethical considerations

Ethical approval was obtained from the Non-Interventional Clinical Research Ethics Committee of Erciyes University (approval no. 621/2021). Institutional permission was obtained from the Erciyes University Health Application and Research Center. Written and verbal consent was obtained from patients willing to participate in the research using the informed consent form. The study was conducted in accordance with the Declaration of Helsinki.

### Statistical analyses

Statistical data analyses were conducted using SPSS 25.0 (Statistical Package for Social Science). Descriptive statistics were reported as frequency (*n*) and percentage (%). Independent sample *t*-tests were utilized to compare differences in scale scores among demographic characteristics for binary groups. At the same time, one-way analysis of variance (ANOVA) was employed for comparisons among more than two groups. Data were evaluated at a significance level of *p* < 0.05 with a 95% confidence interval.

## Results

83.9% of the patients were female, the mean age was 44.653 ± 14.166, and the mean body mass index was 28.618 ± 6.072. 48.0% of the patients were primary or secondary school graduates, 82.7% were married, and most were unemployed. 62.5% of the patients did not have a chronic disease, and 80.8% were non-smokers. In addition, 64.4% of the patients had a previous surgical procedure, and 83.7% were in the ASA II classification. 71.2% of the patients had a surgical procedure that lasted between 2 and 4 h. Laboratory results revealed that the mean calcium levels before and after surgery were 9.617 ± 0.506 and 8.530 ± 0.650, respectively. The preoperative mean vitamin D level was 12.590 ± 6.899 (Table 1).

**Table 1:** Descriptive characteristics of the patients (*n* 104)

	*n* (%)
Gender	
Female	84 (83.9)
Male	20 (16.1)
Mean age (years)	44.653 ± 14.316
Mean BMI	28.618 ± 6.072
Education	
Non-literate	12 (11.7)
Primary school	50 (48.0)
High school	32 (30.7)
University	10 (9.6)
Marital status	
Married	86 (82.7)
Single	18 (17.3)
Occupation	
Worker	5 (4.8)
Officer	10 (9.6)
Retired	6 (5.8)
Unemployed	83 (79.8)
Chronic diseases	
Yes	39 (37.5)
No	65 (62.5)
Smoking	
Yes	20 (19.2)
No	84 (80.8)
Had surgery before	
Yes	67 (64.4)
No	37 (35.6)
ASA classification	
ASA I	13 (12.5)
ASA II	87 (83.7)
ASA III	4 (3.8)
ASA IV	-
Duration of surgery (hour)	
0–2	3 (2.9)
> 2–4	74 (71.2)
> 4–6	20 (19.2)
> 6	7 (6.7)
Laboratory results	
Hypocalcemia	
Yes	25 (24)
No	79(76)
Average calcium levels	
Preoperative	9.617 ± 0.506
Postoperative	8.530 ± 0.650
Average vitamin D level	
Preoperative	12.590 ± 6.899
Total	104

**Fig. 1:**
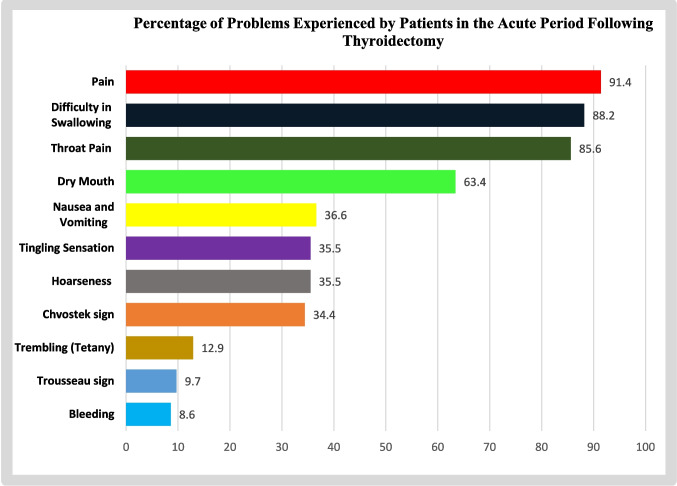
Graph of problems experienced by patients in the acute period following thyroidectomy

When examining issues arising within the first 24 h postoperatively, 37.5% of the patients experienced moderate throat pain, 15.1% had moderate hoarseness, 36.6% faced moderate difficulty in swallowing, and 31.2% experienced mild dry mouth. Furthermore, postoperatively, 37.7% of patients reported mild pain, and 19.4% experienced mild nausea and vomiting. Bleeding occurred in 8.6% of cases, tingling sensation in 35.5%, and trembling in 12.9%. Chvostek and Trousseau signs were identified in 34.4% and 9.7% of cases, respectively. When the pre-and postoperative Richard Campbell Sleep Scale scores of the patients were examined, it was seen that the pre-operative scale average was 70.078 ± 2.801, and the postoperative scale average was 40.723 ± 2.669 points (Fig. 1, Table 2).

**Table 2:** Problems experienced by patients in the acute period following thyroidectomy

Problems	*n* (%)
Throat pain	
Absent	15 (14.4)
Mild	21 (20.2)
Moderate	39 (37.5)
Severe	29 (27.9)
***X*** ± SD	1.788 ± 1.011
Hoarseness	
Absent	60 (64.5)
Mild	13 (14.0)
Moderate	14 (15.1)
Severe	6 (6.5)
***X*** ± SD	0.586 ± 0.941
Difficulty in swallowing	
Absent	11 (11.8)
Mild	23 (24.7)
Moderate	34 (36.6)
Severe	25 (26.9)
***X*** ± SD	1.826 ± 0.999
Dry mouth	
Absent	34 (36.6)
Mild	29 (31.2)
Moderate	16 (17.2)
Severe	14 (15.1)
***X*** ± SD	1.067 ± 1.054
Pain	
Absent	8 (8.6)
Mild	35 (37.7)
Moderate	33 (35.5)
Severe	17 (18.2)
***X*** ± SD	4.163 ± 2.477
Nausea and vomiting	
Absent	59 (63.4)
Mild	18 (19.4)
Moderate	6 (6.5)
Severe	10 (10.8)
***X*** ± SD	1.538 ± 2.797
Bleeding	
No	85 (91.4)
Yes	8 (8.6)
Tingling sensation	
No	60 (64.5)
Yes	33 (35.5)
Trembling (tetany)	
Yok	81 (87.1)
Var	12 (12.9)
Chvostek sign	
No	61 (65.6)
Yes	32 (34.4)
Trousseau sign	
No	84 (90.3)
Yes	9 (9.7)
Preoperative Richard Campbell Sleep Scale	
***X*** ± SD	70.078 ± 2.801
Predischarge Richard Campbell Sleep Scale	
***X*** ± SD	40.723 ± 2.669
Total	104

**Fig. 2:**
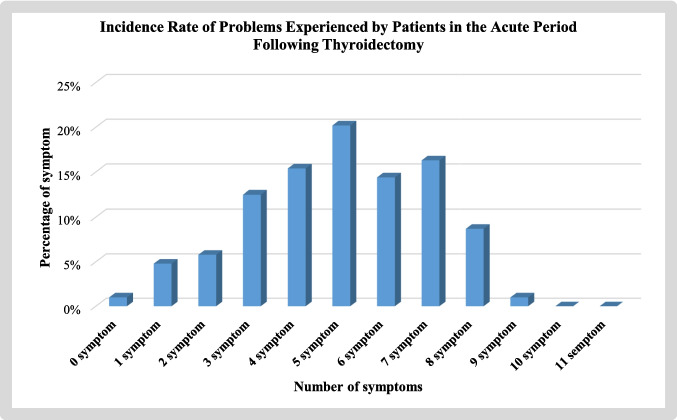
Graph of the incidence rate of problems experienced by patients in the acute period following thyroidectomy

When examining the incidence rates of postoperative symptoms in patients, 20.2% had five symptoms (Fig. 2). When comparing the postoperative RCSQ scale averages based on the problems experienced by patients in the acute period following thyroidectomy, statistically significant differences were found between RCSQ scale averages and throat pain, difficulty swallowing, pain, Chvostek sign, and Trousseau sign (*p* < 0.05). However, no statistically significant differences were observed between RCSQ scale averages and hoarseness, dry mouth, nausea-vomiting, bleeding, tingling sensation, and trembling (*p* > 0.05) (Table 3).

**Table 3:** Comparison of predischarge RCSQ score average according to the problems experienced by patients in the acute period following thyroidectomy (*n* = 104)

Problems	*X* ± SD	Predischarge RCSQ score average
Throat pain		
Absent	70.746 ± 2.243^a^	
Mild	70.628 ± 2.746^a^	*p* = 0.002**
Moderate	70.774 ± 2.558^a^	***F* = 5.453**
Severe	50.400 ± 2.833^b^	
Hoarseness		
Absent	70.234 ± 2.731	
Mild	70.293 ± 3.125	*p* = 0.350
Moderate	60.960 ± 3.018	*F* = 1.107
Severe	50.100 ± 2.141	
Difficulty in swallowing		
Absent	60.523 ± 2.735^a^	
Mild	40.695 ± 2.169^a,b^	*p* = 0.013**
Moderate	40.940 ± 2.963^a,b^	***F* = 3.767**
Severe	30.729 ± 2.235^b^	
Dry mouth		
Absent	70.545 ± 2.464	
Mild	70.258 ± 3.209	*p* = 0.280
Moderate	60.094 ± 2.217	*F* = 1.296
Severe	60.685 ± 3.325	
Pain		
No	70.675 ± 1.743	*p* = 0.004*
Yes	70.029 ± 2.872	**F = 8.628**
Nausea and vomiting		
No	70.375 ± 2.681	*p* = 0.190
Yes	60.563 ± 2.964	F = 1.739
Bleeding		
No	70.237 ± 2.772	*p* = 0.608
Yes	50.175 ± 2.582	t = 0.264
Tingling sensation		
No	70.315 ± 2.710	*p* = 0.365
Yes	60.611 ± 2.957	t = 0.829
Trembling (tetany)		
Yok	70.195 ± 2.797	*p* = 0.684
Var	60.328 ± 2.812	t = 0.167
Chvostek sign		
No	70.411 ± 2.611	*p* = 0.005*
Yes	60.450 ± 3.069	**t = 0.857**
Trousseau sign		
No	70.246 ± 2.832	*p* = 0.004*
Yes	50.500 ± 1.955	**t = 8.458**

*Student’s t-test. **One-way ANOVA

## Discussion

Thyroid surgery is one of the most frequently performed surgeries with low mortality and varied morbidity. Although the rate of complications after thyroidectomy is low, complications such as postoperative bleeding, hypocalcemia, airway problems, vocal cord paralysis, infection, hemorrhage, and hematoma have been reported [19].

In the study, it was observed that the highest prevalence of thyroidectomy was in females (83.9%). Studies in the literature have shown that thyroidectomy surgery is performed similarly at a rate of 71–85%, primarily in women [20–25]. As evidenced by the study results, thyroidectomy prevalence is generally higher in females than males.

The study observed a significant decrease in the preoperative calcium level, with hypocalcemia detected in 24% of the patients. Chvostek’s sign was positive in 34.4% of the patients, and Trousseau’s was positive in 9.7%. Tingling was observed in 35.5% and tremors (tetany) in 12.9% due to low serum calcium levels. In the study, which evaluated early and late complications after thyroidectomy, hypocalcemia was determined to be the most common complication, with a rate of 54.4% [20]. Another study reported the mean postoperative calcium level as 9.31 mg/dl on the first day and 8.21 (4.8–9.9) on the second day [26]. Temporary hypocalcemia was detected in 28% of the patients included in the study. These findings underscore the prevalence and urgency of addressing hypocalcemia in postoperative care.

Statistical analysis revealed significant differences in factors affecting hypocalcemia, including female gender, preoperative diagnosis, intraoperative parathyroid damage, and small nodules with low vitamin D levels. In the study by Yabanoğlu et al. (2019) [23], transient hypocalcemia was detected in ten patients (6.8%) and permanent hypocalcemia in two patients (1.4%) after surgery. In a prospective study, hypoglycemia was observed in 70% of cases. No significant differences were observed in terms of demographic data, malignancy diagnosis, thyroid size, 25(OH)D status, previous treatment, and neck dissection with hypocalcemia. At the end of the study, it was stated that postoperative hypocalcemia remains the most common complication, and the process of prevention and treatment is still not adequately evaluated [26]. Although the percentage of hypocalcemia varies according to different parameters in the literature, it continues to be the most common problem after surgery.

When examining problems occurring within the first 24 h after surgery, it was found that patients experienced pain, difficulty swallowing, throat pain, dry mouth, nausea/vomiting, tingling and hoarseness, Chvostek’s sign, tremors (tetany), Trousseau’s sign, and bleeding. In studies investigating early and late complications after thyroidectomy, investigating early and late complications after thyroidectomy, hoarseness was observed in 33.3% of patients, difficulty swallowing in 32.8%, and hematoma formation in 0.53% of patients in another study [5, 20]. Kwon et al. reported no bleeding in any patients after thyroidectomy, while transient recurrent laryngeal nerve palsy occurred in 11.5% and permanent in 2.3% [27].

In the study by Reizian et al. (2023) [28] examining postoperative pain, all patients in the control group and 90% of patients in the intervention group experienced neck pain within the first 24 h. This was observed in 100% and 83.3% of patients before discharge. One week after surgery, neck pain was observed in 83.3% of the intervention group and most of the control group (90%). In another study examining pain, it was reported that the median pain score for patients was 5, with 53% of them transitioning to chronic pain [29]. In an experimental study investigating the effect of neck exercises on postoperative pain conducted by Türkmen et al. (2022) [30], the mean pain score on the first day after surgery was 2.20 ± 2.22 for the intervention group and 3.00 ± 2.10 for the control group, with no statistically significant difference found.

Before surgery, the average Richard Campbell Sleep Questionnaire (RCSQ) score for patients was 70.078, which decreased to 40.723 predischarge. A statistically significant difference was found between the post-thyroidectomy RCSQ scale averages and throat pain, difficulty swallowing, pain, Chvostek’s sign, and Trousseau’s sign (*p* < 0.05). However, no statistically significant difference was observed between postoperative RCSQ scale averages and hoarseness, dry mouth, nausea-vomiting, bleeding, tingling, and tremors (*p* > 0.05). In the study conducted by Koo et al. (2022) [13] evaluating the preoperative sleep quality of patients undergoing thyroidectomy surgery, it was found that 35 patients (76.1%) had poor preoperative sleep quality. Postoperatively, it was determined that PSQI scores at 1, 4, and 10 months were significantly lower compared to pre-operative scores (*p* < 0.001). Patients experienced sleep disturbances for at least 10 months before and after surgery. The PSQI scores over 5 years post-surgery were significantly decreased compared to pre-operative scores (*p* < 0.001).

La et al. (2117) [31] compared sleep quality in patients undergoing parathyroidectomy and thyroidectomy and found that parathyroid patients had worse pre-operative PSQI scores than thyroid patients. Before surgery, a more considerable proportion of parathyroid patients exhibited poor sleep quality (PSQI score > 5) compared to the thyroid group (69% vs. 51%). Another study observed that on the first day postoperatively, 40% of patients experienced sleep problems, 100% experienced pain, 95% experienced respiratory problems, 98.3% experienced eating and drinking issues, and 83.3% experienced communication problems. By the end of the first week, these percentages decreased to 3.3%, 28.3%, 13.3%, 18.3%, and 21.7%, respectively. Patients experienced problems up to 4 weeks postoperatively, indicating that they faced challenges, particularly on the first day and first week after surgery [32].

## Conclusion

The study revealed that the most common problems after thyroidectomy were pain, difficulty swallowing, and sore throat. The sleep quality scale score average decreased in the postoperative period compared to the pre-operative period. The study revealed that sore throat, difficulty swallowing, pain, Chvostek sign, and Trousseau sign reduced sleep quality. Surgical clinic nurses must be aware of the role of early problems and their impact on sleep to monitor and evaluate the clinical course of thyroidectomy patients closely. The research findings show that thyroidectomy patients should be included in routine follow-up plans even after discharge from the hospital. In addition, patients should be counseled about possible complications in the preoperative period.

## Strengths and limitations of the study

The weaknesses of the study include the fact that it was conducted in a single center and the fact that the data collection process took a long time. In addition, few studies on thyroidectomy surgery focus on a single symptom, and most of them are retrospective. One of the strengths of this study, which we think is very important in terms of data obtained from a prospective study, is that there is no similar study in the literature and that it reveals the early results of patients who underwent thyroidectomy surgery.

## Data Availability

No datasets were generated or analysed during the current study.
